# Demographic, clinical and laboratory differences between paediatric acute COVID-19 and PIMS-TS—results from a single centre study in the UK

**DOI:** 10.3389/fped.2023.1219654

**Published:** 2023-11-10

**Authors:** Prince Jiju, Michail Matalliotakis, Steven Lane, Waison Wong, Christian M. Hedrich, Clare E. Pain

**Affiliations:** ^1^ Department of Paediatric Medicine, Alder Hey Children’s NHS Foundation Trust, Liverpool, United Kingdom; ^2^ Department of Paediatric Rheumatology, Alder Hey Children’s NHS Foundation Trust, Liverpool, United Kingdom; ^3^ Department of Biostatistics, Institute of Infection, Veterinary and Ecological Sciences, University of Liverpool, Liverpool, United Kingdom; ^4^ Department of Paediatric Infectious Diseases and Immunology, Alder Hey Children’s NHS Foundation Trust, Liverpool, United Kingdom; ^5^ Department of Women’s and Children’s Health, Institute of Life Course and Medical Sciences, University of Liverpool, Liverpool, United Kingdom

**Keywords:** acute COVID-19 children, MIS-C multisystem inflammatory syndrome in children, PIMS-TS, retrospective study, epidemiology, clinical feature

## Abstract

**Background:**

Paediatric symptomatic SARS-CoV-2 infections associate with two presentations, acute COVID-19 and paediatric inflammatory multisystem syndrome temporally associated with SARS-CoV-2 (PIMS-TS). Phenotypic comparisons, and reports on predictive markers for disease courses are sparse and preliminary.

**Methods:**

A chart review of COVID-19 and PIMS-TS patients (≤19 years) admitted to Alder Hey Children's NHS Foundation Trust, a tertiary centre in the North-West of England, was performed (02/2020–09/2022).

**Results:**

A total of 161 symptomatic COVID-19 and 50 PIMS-TS patients were included. Peaks in admissions of patients with PIMS-TS occurred approximately 4 weeks after those for acute COVID-19. The incidence of in-patients with PIMS-TS reduced over time, and there were no admissions after February 2022. When compared to acute COVID-19, PIMS-TS patients were older (median: 10.3 years vs. 2.03 years; *p* < 0.001). There were no differences in gender distribution, but minority ethnicities were over-represented among PIMS-TS patients. Regional ethnic distribution was reflected among acute COVID-19 patients (66% vs. 84.5% White Caucasian, *p* = 0.01). Pre-existing comorbidities were more common among acute COVID-19 patients (54.7% vs. 8%, *p* < 0.001). PIMS-TS patients more commonly presented with abdominal symptoms (92% vs. 50.3%), neurological symptoms (28% vs. 10.6%) and skin rashes (72% vs. 16.8%), (*p* ≤ 0.01) when compared with acute COVID-19, where respiratory symptoms were more common (51.6% vs. 32%, *p* = 0.016). PIMS-TS more frequently required intensive care admission (64% vs. 16.8%), and inotropic support (64% vs. 9.3%) (all *p* < 0.05). More deaths occurred among acute COVID-19 patients [0 vs. 7 (4.4%)], with 5/7 (71%) in the context of pre-existing comorbidities. When compared to acute COVID-19, PIMS-TS patients exhibited more lymphopenia and thrombocytopenia, a more pronounced acute phase reaction, and more hyponatraemia (*p* < 0.05). Partial least square discriminant analysis of routine laboratory parameters allowed (incomplete) separation of patients at diagnosis, and variable importance projection (VIP) scoring revealed elevated CRP and low platelets as the most discriminatory parameters.

**Conclusion:**

Admissions for PIMS-TS reduced with increasing seroconversion rates in the region. Young age and pre-existing comorbidities associate with hospital admission for acute COVID-19. While PIMS-TS may present more acutely with increased need for intensive care, acute COVID-19 had an increased risk of mortality in this cohort.

## 1. Introduction

Severe acute respiratory syndrome coronavirus 2 (SARS-CoV-2) is the pathogen responsible for the COVID-19 pandemic (1). Since December 2019, COVID-19 presented in waves that associated with various SARS-CoV-2 variants of concern (VoC) which emerged over time (1, 2). New variants were the result of hypermutation, predominantly affecting the spike region of the virus' RNA genome (1, 3). Because the spike protein is responsible for the infection of host cells, virus variants exhibit variable pathogenicity (3).

Notably, SARS-CoV-2 infections can manifest in a variety of clinical pictures. While most patients, across age groups, are asymptomatic or exhibit mild upper respiratory symptoms, others become severely unwell, particularly those with underlying comorbidities and/or the elderly (4–6). Severe COVID-19 can result in organ failure and death (7, 8). Mortality rates in the United Kingdom (UK) between 2020 and 2021 were 113.8 per 100,000 (9). Mortality was 62 times higher among patients aged >65 years (6).

When compared to adults, children and young people (CYP) infected with SARS-CoV-2 exhibit reduced disease severity and lower mortality, with infections typically being mild or asymptomatic (10). However, a subset of children develop severe acute COVID-19 requiring hospitalisation (11–13). Mortality associated with acute COVID-19 is low in children when compared to adults (estimated 1.83/million CYP in England) (14). Similar to adults, CYP presenting with severe COVID-19 are more likely to have comorbidities (11, 13, 15–17). Pre-existing conditions associated with severe COVID-19 in CYP include chronic lung disease, airway anomalies, neurodevelopmental disorders, cardiovascular disease, prematurity, diabetes mellitus, obesity (13, 15), immunosuppression, and sickle cell disease (16). However, published reports on underlying risk factors for severe acute COVID-19 and hospitalisation in children were descriptive, limited in numbers, and sometimes contradictory.

A subset of CYP present with a post-infectious hyperinflammatory syndrome termed Paediatric Multisystem Inflammatory Syndrome Temporally Associated with SARS-CoV-2 (PIMS-TS) or multisystem inflammatory syndrome in children (MIS-C) (18, 19). PIMS-TS typically presents between two and six weeks after the exposure to SARS-CoV-2 (20). It is characterised by persistent fever, elevated laboratory markers of inflammation, and evidence of single or multi-organ dysfunction (21, 22). PIMS-TS represents a disease spectrum, and mild cases may be missed. The full clinical picture of PIMS-TS frequently includes gastrointestinal and cardiovascular involvement (23–25). In a study in the Czech Republic reporting cases between November 2020 and March 2022, the incidence of PIMS-TS was estimated as 53/100,000 SARS-CoV-2 positive children (26).

This study aimed to compare incidences (over time), clinical and laboratory features of PIMS-TS and acute COVID-19 in CYP admitted to a tertiary paediatric hospital in the North-West of England.

## 2. Methods

### 2.1. Study design and participants

A retrospective chart review was undertaken in CYP (≤19 years) admitted to Alder Hey Children's NHS Foundation Trust, Liverpool, for acute COVID-19 or PIMS-TS between 10/02/2020 to 31/08/2022. The study was approved as a service evaluation by the local audit committee.

### 2.2. Case definitions and ascertainment

Several different diagnostic criteria exist to define PIMS-TS/MIS-C. For this study, the case definition of the Royal College of Paediatrics and Child Health (RCPCH) was used (19). Patients were classified by the centre's PIMS-TS multidisciplinary team (including infectious disease, rheumatology, general paediatric and cardiology specialists).

Acute COVID-19 cases were defined as CYP with an acute hospital admission who tested positive for SARS-CoV-2 by RT-PCR (real time polymerase chain reaction) AND the admission was due to COVID-19. Hospital acquired acute COVID-19 cases were also included if patients tested positive for SARS-CoV-2 AND developed symptoms suggestive of acute COVID-19 during their admission. All in-patients testing positive for SARS-CoV-2 (RT-PCR) between 10/02/2020 to 31/08/2022 were identified from the microbiology database. Cases were screened and case definitions for symptomatic acute COVID-19 were applied by PJ and MM to exclude patients with incidentally positive SARS-CoV-2 RT-PCR (e.g., elective admissions, surgical procedures, admissions for mental health issues). Unclear cases were reviewed by CH, CP, WW.

Electronic medical records of inpatients meeting the definition of PIMS-TS or acute COVID-19 were accessed to record demographic (age, sex, and ethnicity) and clinical information, including date of admission, comorbidities, clinical symptoms and signs, laboratory parameters within 24 h of admission and peak abnormal values (including white blood cells [WBC], neutrophils, lymphocytes, C-reactive protein [CRP], ferritin, triglycerides, platelets, INR, fibrinogen, D-dimer, haemoglobin [Hb], sodium, urea, creatinine, bilirubin, alanine transaminase [ALT], aspartate aminotransferase [AST], and treatment. Disease severity as estimated by recording duration of admission [days], admission to critical care, level of medical intervention required (e.g., fluid resuscitation, inotropic support, non-invasive or invasive ventilatory support), and mortality was recorded.

### 2.3. Statistical analysis

Statistical analyses were performed in Rstudio 2022.07.2 (27), utilising R version 4.2.2 and the “tidyverse” package (28). Quantitative variables were reported using median and interquartile range (IQR), as most variables followed a non-parametric distribution. Continuous variables were tested for normal distribution using Shapiro-Wilk test (*p > 0.05*); *t*-tests were used for comparisons between groups if following normal distribution; Mann–Whitney tests were used for variables not following normal distribution. Categorical variables were compared between groups using Chi-Square tests or Fisher's exact test. Statistical significance was determined as a *p*-value below or equal to 0.05 (*p ≤ *0.05). Bonferroni correction was utilised when comparing clinical characteristics to adjust for multiple comparisons. Patients with missing laboratory data were excluded in the univariate analysis. Laboratory parameters with ≥95% missing data in either group were not compared in univariate or multivariate analysis. A partial least squares-discriminant analysis (PLS-DA) model and a variable importance plot of laboratory tests, were conducted on Rstudio using the “mixOmics” (29) and “tidyverse” package (28). For analysis of admission blood tests, laboratory tests with the fewest missing datapoints (≤16% for either group) were selected, and missing values were imputed by using the mean of the variable for each group. For analysis of peak blood tests, no imputation was done due to a greater percentage of missing datapoints, and any patients with missing values were omitted.

We chose the PLS-DA model, a supervised multivariate statistical method, as a dimensionality reduction tool to produce latent variables, which are a linear combination of the laboratory parameters. 95% confidence ellipses were used to visually estimate whether groups can be separated based on the laboratory parameters. A variable importance plot (VIP) of laboratory parameters was used to display which laboratory variables most contributed to the PLS-DA model. The variable importance plot produces VIP scores for each independent variable in the PLS-DA model, and thus the importance of each variable in contributing to the separation in the two groups of the PLS-DA model. This approach was used to identify which laboratory variables best predict whether patients would belong to the COVID-19 or PIMS-TS group.

## 3. Results

### 3.1. Demographics and epidemiology

During the study period a total of 50 patients met the case definition for PIMS-TS. Over the same interval, 848 CYP (≤19 years) tested positive for SARS-CoV-2 by RT-PCR. Of these, 687 CYP (81%) were asymptomatic and/or incidental and were excluded from the analysis. This left 161 patients who were admitted to hospital due to acute COVID-19 or developed symptomatic disease during hospital admission. Two CYP had two separate acute COVID-19 infections and were included in the analysis twice.

PIMS-TS patients were significantly older (median: 10.3 [IQR: 5.65] vs. 2.03 [IQR: 10.6] years; *p* < 0.001) (Table 1). No differences were recorded in gender distribution. Notably, while in the PIMS-TS cohort ethnic minorities were over-represented, the acute COVID-19 cohort reflected the regional ethnic distribution (63.6% vs. 83.8% White Caucasians, *p* < 0.01), with the White Caucasian ethnic group representing 84% of the regional population (National Census, 2021) (30).

**Table 1:** Demographics of CYP admitted with PIMS-TS and acute COVID-19.

Parameter		PIMS-TS (*n = *50)	Acute COVID-19 (*n = *161)	*p*-value
Median age (Interquartile range, IQR)		10.3 (5.65)	2.03 (10.6)	<0.001
Male to female ratio		1:1	1:0.77	0.418
Ethnicity	White caucasian % (*n*)	63.6 (28/44)	83.8 (119/142)	0.004
	Other % (*n*)	36.4 (16/44)	16.2 (23/142)	
	Not stated % (*n*)	12.0 (6/50)	11.7 (19/162)	
Any comorbidity % (*n*)		4.0 (2/50)	42.2 (68/161)	<0.001
	Neonate (<28 days old) % (*n*)	0.0 (0/50)	6.2 (10/161)	
	Prematurity in infant (<1 year old) % (*n*)	0.0 (0/50)	9.9 (16/161)	
	Neurological & neurodevelopmental % (*n*)	0.0 (0/50)	9.3 (15/161)	
	Cardiovascular % (*n*)	0.0 (0/50)	15.5 (25/161)	
	Metabolic/endocrine % (*n*)	0.0 (0/50)	8.7 (14/161)	
	Respiratory % (*n*)	4.0 (2/50)	9.9 (16/161)	
	Primary immunodeficiency % (*n*)	0.0 (0/50)	1.2 (2/161)	
	Secondary immunodeficiency % (*n*)	0.0 (0/50)	9.9 (16/161)	
	Oncological % (*n*)	0.0 (0/50)	5.0 (8/161)	
	Renal disease % (*n*)	0.0 (0/50)	7.5 (12/161)	
	Liver disease % (*n*)	0.0 (0/50)	1.9 (3/161)	
	Trisomy 21% (*n*)	0.0 (0/50)	1.2 (2/161)	
	Other % (*n*)	0.0 (0/50)	9.9 (16/161)	
	Multiple comorbidities (more than one) % (*n*)	0.0 (0/50)	27.3 (44/161)	* *

Pre-existing comorbidities were more common among acute COVID-19 patients (42.2% vs. 4%, *p* < 0.001), and 28% had more than one comorbidity. Most common pre-existing diseases included cardiovascular disease (15.5%), then prematurity in infant (<1 year old), respiratory and secondary immunodeficiency (all 9.9%) (Table 1, Supplementary Table 1). The two PIMS-TS patients with comorbidities had asthma.

**Figure 1:**
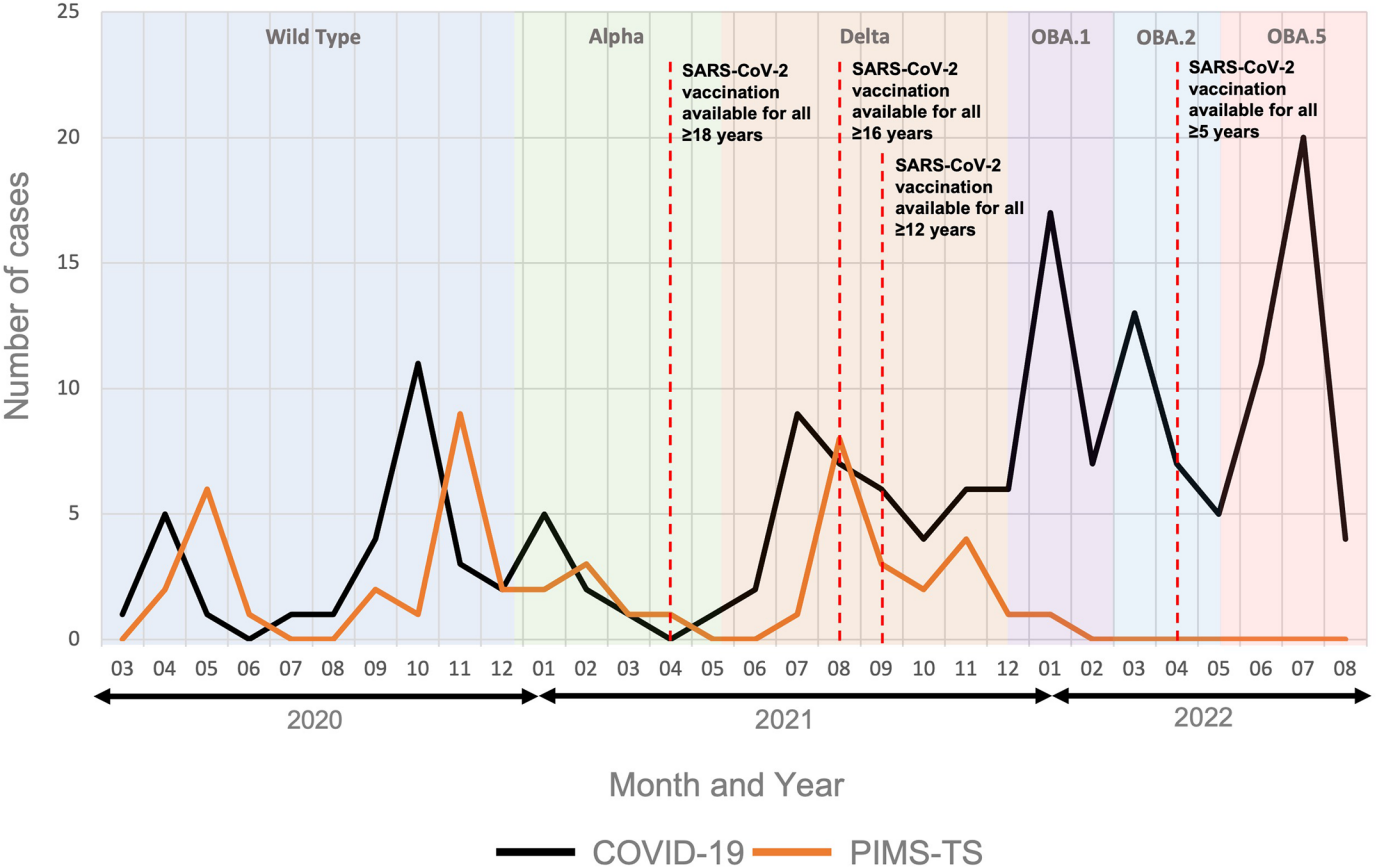
Hospital admissions for PIMS-TS and acute COVID-19. The figure includes data from 50 patients admitted for PIMS-TS and 161 acute COVID-19 patients*.* Predominant SARS-CoV-2 variants in the region are indicated over time. OBA.1: Omicron variant BA1; OBA.2: Omicron variant BA2; OBA.5: Omicron variant BA5 (31). Timelines of the SARS-CoV-2 vaccination roll out are indicated (32).

As previously reported, hospital admissions for PIMS-TS lagged approximately 4 weeks behind those for acute COVID-19 (Figure 1) (20, 33). No cases of PIMS-TS were recorded from February 2022, which coincided with the predominance of the SARS-CoV-2 Omicron variant (31).

### 3.2. Clinical presentation and disease severity

As per case definition, all PIMS-TS patients exhibited fevers during admission which compared to 72% of acute COVID-19 patients (*p* < 0.001) (Table 2). PIMS-TS patients more frequently presented with gastrointestinal symptoms (all *p* < 0.001), neurological symptoms (28% vs. 10.6%, *p* = 0.0023) and/or headaches (52 vs. 12.4%, *p* < 0.001). Furthermore, PIMS-TS patients more commonly exhibited symptoms also associated with “historic” Kawasaki disease. Patients admitted with acute COVID-19 more frequently exhibited upper respiratory symptoms (51.6% vs. 32%, *p* = 0.015). A total of 27 (16.8%) acute COVID-19 patients were admitted with feeding concerns, while no PIMS-TS patients were (*p* = 0.001).

**Table 2:** Clinical phenotype of PIMS-TS and acute COVID-19 patients.

Clinical features		PIMS-TS (*n* = 50)	Acute COVID-19 (*n* = 161)	*p*-value
Gastrointestinal	Abdominal pain % (*n*)	82.0 (41/50)	19.3 (31/161)	<0.001*
	Diarrhoea % (*n*)	56.0 (28/50)	24.2 (39/161)	<0.001*
	Nausea/Vomiting % (*n*)	72.0 (36/50)	34.8 (56/161)	<0.001*
Respiratory	Upper respiratory tract symptoms or signs % (*n*)	32.0 (16/50)	51.6 (83/161)	0.015*
	Signs of increased work of breathing % (*n*)	24.0 (12/50)	31.7 (51/161)	0.3
Cardiovascular	Tachycardic on admission % (*n*)	62.0 (31/50)	52.2 (84/161)	0.22
Neurological	Confusion or neurological concern % (*n*)	28.0 (14/50)	10.6 (17/161)	0.0023*
	Headache % (*n*)	52.0 (26/50)	12.4 (20/161)	<0.001*
Bilateral non-purulent conjunctivitis % (*n*)		76.0 (38/50)	1.2 (2/161)	<0.001
Skin rash % (*n*)		72.0 (36/50)	16.8 (27/161)	<0.001
Mucous membrane changes % (*n*)		56.0 (28/50)	0.0 (0/161)	<0.001
Swelling to hands/feet % (*n*)		42.0 (21/50)	3.1 (5/161)	<0.001
Cervical lymphadenopathy % (*n*)		30.0 (15/50)	3.7 (6/161)	<0.001
Hepatosplenomegaly % (*n*)		8.0 (4/50)	5.6 (9/161)	0.53
Arthritis % (*n*)		6.0 (3/50)	0.0 (0/161)	0.002
Lethargy % (*n*)		6.0 (3/50)	12.4 (20/161)	0.203
Feeding concerns % (*n*)		0.0 (0/50)	16.8 (27/161)	0.0019
Fever on admission % (*n*)		84.0 (42/50)	59.6 (96/161)	0.0016
Fever at any point during admission % (*n*)		100.0 (50/50)	72.0 (116/161)	<0.001

* These comparisons remained significant after correcting using the Bonferroni method when making multiple comparisons within organ systems. *p*-values presented are before Bonferroni adjustment. No correction was applied to independent comparisons.

To assess disease severity, admission to critical care, cardiorespiratory supportive therapy and duration of hospital admissions were compared (Table 3). Patients with PIMS-TS more frequently required oxygen supplementation and inotropic support (64% vs. 9.3%, *p* < 0.001) but no significant differences were observed in escalating respiratory support via non-invasive (NIV) or invasive ventilation. A higher proportion of critical care admissions occurred in the PIMS-TS when compared to the acute COVID-19 cohort (64% vs. 16.8%, *p* < 0.001). No PIMS-TS patients required extracorporeal membrane oxygenation (ECMO) whereas 3 (1.9%) patients with acute COVID-19 did (*p* = 0.3). PIMS-TS patients had a longer median hospitalisation (8 vs. 3 days, *p* < 0.001). Seven patients admitted with acute COVID-19 died, while all PIMS-TS patients in this cohort recovered (Box 1).

**Table 3:** Parameters of disease severity.

Parameters of disease severity		PIMS-TS (*n = *50)	Acute COVID-19 (*n = *161)	*p*-value
Respiratory	Oxygen requirement % (*n*)	48.0 (24/50)	29.8 (48/161)	0.017*
	Non-invasive ventilation % (*n*)	4.0 (2/50)	7.5 (12/161)	0.39
	Invasive ventilation % (*n*)	12.0 (6/50)	14.3 (23/161)	0.68
Cardiovascular	Hypotensive requiring fluid resuscitation % (*n*)	68.0 (34/50)	19.9 (32/161)	<0.001*
	Inotropic support % (*n*)	64.0 (32/50)	9.3 (15/161)	<0.001*
Median hospital admission time (days)		8.0	3.0	<0.001
Admission to critical care % (*n*)		64.0 (32/50)	16.8 (27/161)	<0.001
Extracorporeal membrane oxygenation (ECMO) requirement % (*n*)		0.0	1.9 (3/161)	0.33
Renal replacement therapy % (*n*)		2.0 (1/50)	2.5 (4/161)	0.84
Patient mortality % (*n*)		0.0	4.4 (7/161)	0.23

* These comparisons remained significant after correcting using the Bonferroni method when making multiple comparisons within organ systems. *p*-values presented are before Bonferroni adjustment. No correction was applied to independent comparisons.

BOX 1 **Acute COVID-19 cases with fatal outcomes.**

Most patients in this cohort who died due to acute COVID-19 were girls (5/7, 71%), with it appearing that SARS-CoV-2 Delta variant was the most common (5 Delta vs 2 Omicron variant). Only 1/7 had SARS-CoV-2 variant testing (confirming Delta variant), with the remaining 6/7 assumed their variant using the most prevalent VoC in the North-West at time of positive SARS-CoV-2 RT-PCR test. Patient age range varied from 7 days to 13 years old, and 5/7 (71%) had comorbidities:

• 2 premature neonates born at 32 and 35 weeks;
• 1 infant with cardiomyopathy;
• 4 school-aged children: two did not have pre-existing comorbidities (one died of cerebral haemorrhage, the other one with ARDS secondary to COVID-19, leading to necrotising pneumonia with empyema and consequently multi-organ failure), one with complex neuro-oncological disability (midline glioma) and one with metabolic (mitochondrial storage) disease.

Four children died due to respiratory deterioration, one due to cardiovascular, one with neurological deterioration and one with multi-organ failure. Six of the seven children received mechanical ventilation and cardiovascular support with inotropes. Two children received extracorporeal membrane oxygenation (ECMO).

### 3.3. Laboratory phenotype and predictive markers

PIMS-TS patients exhibited higher systemic markers of inflammation when compared to acute COVID-19 patients, including leucocytosis (*p* = 0.01), neutrophilia and elevated CRP (*p* < 0.001) (Table 4). While ferritin (*p* = 0.01), fibrinogen (*p* < 0.001) and D-dimers (*p* = 0.028) were higher in PIMS-TS patients, they were infrequently tested in acute COVID-19. PIMS-TS patients exhibited increased overall WBC but more frequent lymphocytopenia (*p* < 0.001) and thrombocytopenia (*p* < 0.001). Patients with PIMS-TS more frequently exhibited hyponatraemia (*p* < 0.001).

**Table 4:** Laboratory test results at admission.

Blood tests	PIMS-TS Median (IQR)	PIMS-TS *%,* (*n*) *n = *50^a^	Acute COVID-19 Median (IQR)	Acute COVID-19 *%,* (*n*) *n = *161^a^	*p*-value
White blood cells (WBC) ×10^9^/L	9.75 (11.1)	100 (50/50)	7.94 (5.44)	85.1 (137/161)	0.01
Neutrophils ×10^9^/L	7.68 (9.18)	100 (50/50)	3.8 (5.1)	85.1 (137/161)	<0.001
Lymphocytes ×10^9^/L	0.795 (0.808)	100 (50/50)	2.07 (2.22)	84.5 (136/161)	<0.001
C-reactive protein (CRP) mg/L	206.0 (114)	100 (50/50)	4.0 (12)	84.5 (136/161)	<0.001
Ferritin ng/ml	781.0 (1,196)	96.0 (48/50)	324.0 (718)	9.3 (15/161)	0.01
Triglycerides mmol/L	1.8 (1.3)	86.0 (43/50)	0.96 (0.605)	6.8 (11/161)	0.002
Platelets ×10^9^/L	138.0 (86.5)	100 (50/50)	313.0 (190)	84.5 (136/161)	<0.001
INR	1.14 (0.153)	100 (50/50)	1.17 (0.265)	29.8 (48/161)	0.49
Fibrinogen g/L	5.0 (1.74)	100 (50/50)	2.4 (1.12)	18.6 (30/161)	<0.001
D-dimer ng/ml	3,610.0 (3,158)	96.0 (48/50)	1,758.0 (3,402)	9.3 (15/161)	0.028
Haemoglobin (Hb) g/L	116.0 (26.8)	100 (50/50)	116.0 (21)	85.1 (137/161)	0.72
Sodium mmol/L	133.0 (5)	100 (50/50)	138.0 (3)	87.0 (140/161)	<0.001
Urea mmol/L	4.85.0 (5.12)	100 (50/50)	3.4 (2.3)	87.0 (140/161)	<0.001
Creatinine mmol/L	51.0 (39.8)	100 (50/50)	32.0 (21.5)	87.0 (140/161)	<0.001
Bilirubin µmol/L	8.0 (6)	100 (50/50)	7.0 (9)	53.4 (86/161)	0.43
Alanine transaminase (ALT) iu/L	28.5 (34.5)	100 (50/50)	25.0 (29)	53.4 (86/161)	0.6
Aspartate aminotransferase (AST) iu/L	29.0 (31)	100 (50/50)	35.0 (27)	53.4 (86/161)	0.22

^a^ Percentage and number of populations which underwent these laboratory tests on admission.

**Figure 2:**
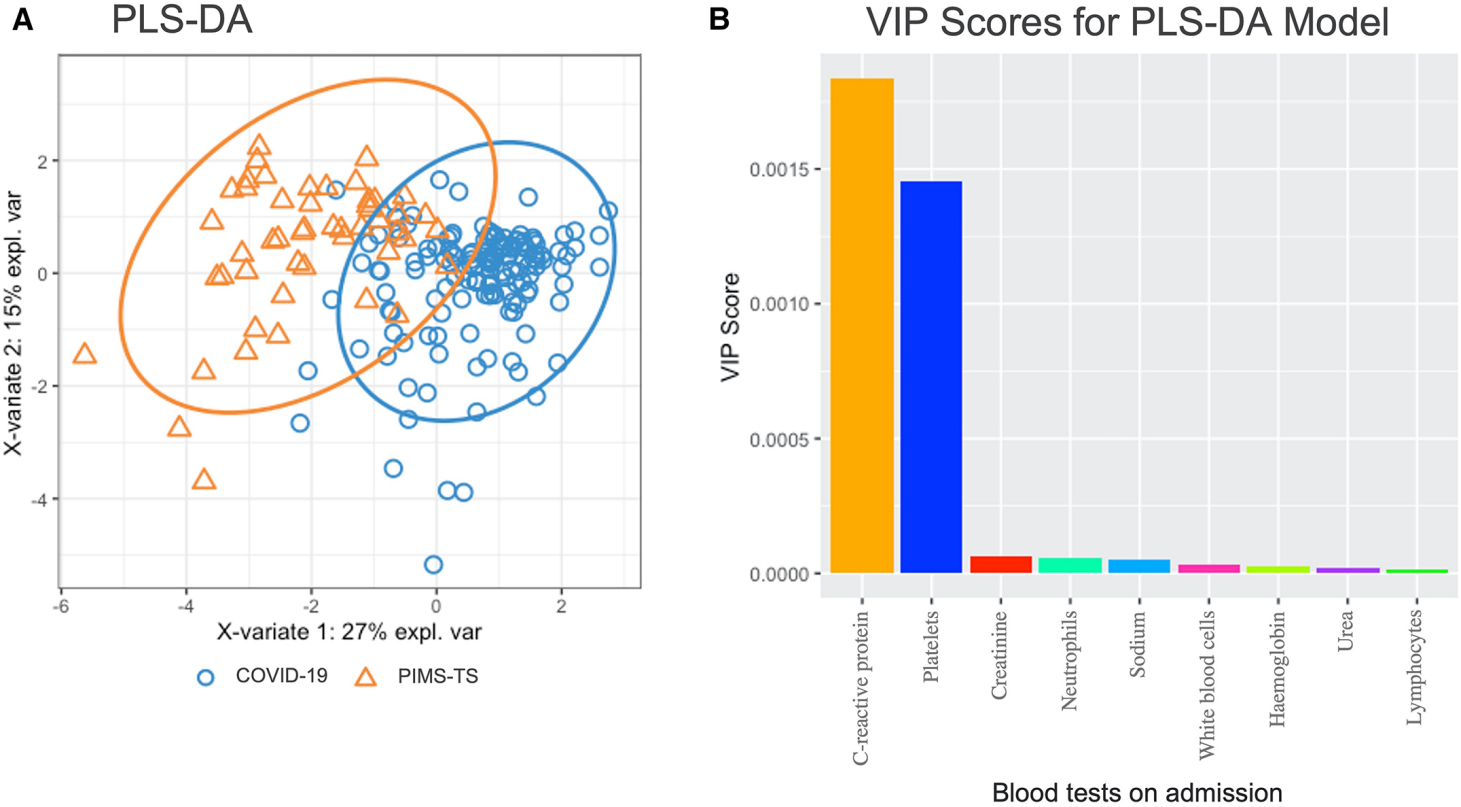
Partial least squares-discriminant analysis (PLS-DA) in PIMS-TS (*n = *50) and acute COVID-19 (*n = *161) patients at admission. Blood tests included were haemoglobin, platelets, white blood cells, neutrophils, lymphocytes, C-reactive protein, sodium, urea, creatinine. (**A**) PLS-DA analysis showing individual samples with the confidence ellipses of PIMS-TS and COVID-19. (**B**) VIP analysis showing variable importance plots of blood tests included in the PLS-DA model.

To investigate whether laboratory tests at admission differentiate PIMS-TS from acute COVID-19 patients, partial least-squares discriminant analysis (PLS-DA) was performed, including the following parameters: haemoglobin, platelets, white blood cells, neutrophils, lymphocyte counts, CRP, sodium, urea, and creatinine (Figure 2). While PLS-DA did not completely separate PIMS-TS from acute COVID-19 patients, variable importance projection (VIP) analysis showed that CRP elevation and reduced platelet numbers were more pronounced in PIMS-TS as compared to acute COVID-19 patients.

Considering peak changes of laboratory parameters, overall, PIMS-TS patients exhibited a more pronounced acute phase reaction when compared to COVID-19 infection (elevated WBC, neutrophils, CRP, fibrinogen) (Supplementary Table 2). However, features overlapped more than at admission and did not allow for clear separation between groups (Supplementary Figure 1). While overlapping significantly between groups, VIP analysis suggested elevated CRP and thrombopenia to be associated with PIMS-TS more than active COVID-19.

### 3.4. Treatment of PIMS-TS and COVID-19

Various drug therapies were used to treat PIMS-TS and acute COVID-19 (Table 5). The most common treatments in PIMS-TS included intravenous immunoglobulins (IVIG) (82%, 41/50) and methylprednisolone (76%, 38/50) Dexamethasone was prescribed in 14.9% (23/161) of acute COVID-19 patients. Furthermore, 16% (8/50) of PIMS-TS patients received biologic disease modifying anti-inflammatory therapy, which compared to 3.7% of acute COVID-19 patients. Treatments included the recombinant interleukin receptor antagonist anakinra (8%) and the IL-6 receptor antagonist tocilizumab (8%).

**Table 5:** Comparison of treatment in patients with PIMS-TS and acute COVID-19.

Treatment		PIMS-TS % (*n*)	Acute COVID-19 % (*n*)
Low dose aspirin (3–5 mg/kg/day)		96.0 (48/50)	0.6 (1/161)
High dose aspirin (30–50 mg/kg/day)		66.0 (33/50)	0.0 (0/161)
Intravenous immunoglobulin (IVIG)		82.0 (41/50)	1.9 (3/161)
Intravenous methylprednisolone		76.0 (38/50)	2.5 (4/161)
Tapering oral prednisolone following methylprednisolone		74.0 (37/50)	1.9 (3/161)
Dexamethasone		4.0 (2/50)	14.3 (23/161)
Biologics		16.0 (8/50)	3.7 (6/161)
	Anakinra	8.0 (4/50)	2.5 (4/161)
	Tocilizumab	8.0 (4/50)	1.2 (2/161)
Remdesivir		0.0 (0/50)	10.6 (17/161)
SARS-CoV-2 specific monoclonal antibodies		0.0 (0/50)	3.1 (5/161)
	Sotrovimab	0.0 (0/50)	2.5 (4/161)
	Ronapreve	0.0 (0/50)	0.6 (1/161)
Antimicrobials (intravenous)		94.0 (47/50)	58.4 (94/161)

Remdesivir, a viral RNA polymerase inhibitor, was used in 10.6% of acute COVID-19 patients. Four patients (2.5%) received the recombinant anti-SARS-CoV-2 monoclonal antibody Sotrovimab and 1 patient (0.6%) received Ronapreve, a combination of anti-SARS-CoV-2 recombinant antibodies casirivimab and imdevimab. Almost all PIMS-TS patients (94%) received intravenous antimicrobial therapy, compared to 58.4% of acute COVID-19 patients (*p* < 0.001).

## 4. Discussion

This single centre retrospective study from a large tertiary children's hospital in the North-West of England, which provides healthcare to over 330,000 children and young people each year (34), reports varying proportions of COVID-19 and PIMS-TS patients over time. While PIMS-TS was the dominant cause of SAR-CoV-2 associated hospital admissions early in the pandemic, cases of admissions due to acute COVID-19 rose sharply from January 2022 with the emergence of the Omicron variant in the region. This coincided with a decline in hospitalisations for PIMS-TS. The incidence of admissions for SARS-CoV-2 associated disease at Alder Hey Hospital follows the pattern seen nationally with corresponding spikes in infection and admission at similar times (35). Several groups previously suggested decreasing numbers of PIMS-TS cases with the Delta (20, 36–38) and especially the Omicron variant (20, 36–40), despite a significant rise in the number of hospitalised acute COVID-19 cases. Notably, in contrast to some of these reports, we observed an increase in both acute COVID-19 and PIMS-TS cases with the Delta variant (41).

Two main theories exist on why PIMS-TS numbers declined with the Delta and/or the Omicron variant:

• One hypothesis suggests that increasing SARS-CoV-2 seroprevalence, through infection or vaccination, may have reduced the PIMS-TS risk (39, 42, 43). However, Sorg et al. suggested that vaccination did not significantly alter the PIMS-TS risk in Germany because all age groups, including those not eligible for vaccination, had falling rates of PIMS-TS (37). In the here presented study, it was difficult to capture whether children had been vaccinated due to a lack of documentation in medical records. However, a significant proportion of the study population were likely not vaccinated because, in the UK, vaccination programmes for 12- to 15-year-olds only began in September 2021, and for 5- to 11-year-olds in April 2022. Furthermore, vaccination rates in the North-West were the lowest in England (44). Notably, in the here reported cohort and others (23, 45, 46), children admitted for PIMS-TS were older when compared to those admitted for COVID-19 (median: 10.3 vs. 2.03 years). This may suggest increased risk for PIMS-TS in individuals that first encounter SARS-CoV-2 in their school-age years as compared to younger children. Vaccination or infection early in life, which results in the acquisition of immune memory and an orchestrated innate and adaptive immune response against SARS-CoV-2, however, may prevent the development of PIMS-TS upon re-exposure later in life. Increasing and then constantly high case numbers among children in the region resulted in an estimated 93% of children having experienced SARS-CoV-2 infection by September 2022 (unpublished, UK Health Security Agency, UKHSA). This may explain the absence of admissions for PIMS-TS in the region after February 2022. Because vaccination may offer even better protection from PIMS-TS (including strong T cell responses) (47, 48), the observed disappearance of PIMS-TS over time may demonstrate the success of vaccination programmes, preventing hospitalisation and complications. Vaccination not only reduced COVID-19 severity (49), but may also have conferred protection against PIMS-TS (50–52). Therefore, a cornerstone of future public health planning in the face of disease outbreaks should focus on ensuring vaccine uptake remains high.
• A second theory claims that mutations in the virus' spike protein may affect infectivity, virulence, and disease outcomes (3, 53, 54). Reducing rates of PIMS-TS during the pre-vaccine Delta period in some studies suggests that changes to the SARS-CoV-2 virus may have been responsible. However, observations from the here presented study, namely increasing numbers of both COVID-19 and PIMS-TS during the “Delta variant wave”, argue for increasing seroconversion through infection or vaccination having contributed to reduced numbers of PIMS-TS patient cases. Initially, during the first waves with the wild-type and alpha variants, children may have been less affected because of reduced travel activity, shielding, school closures in the regions and reduced infectivity and virulence within the age group (55–57). The proportion of PIMS-TS (as compared to COVID-19) were relatively high throughout the “Wild-type”, “Alpha” and “Delta waves”. Across the UK, approximately 40% of 5- to 11-year-old and 82% of 12- to 18-year-old children and young people had detectable SARS-CoV-2 antibodies by December 2021, the end of the “Delta wave” (58). In adults, at a similar time point, >95% tested positive for SARS-CoV-2 antibodies (59). Of the 82.4% of 12- to 18-year-olds who tested positive for SARS-CoV-2 antibodies, less than half (43.1%) had received at least one COVID-19 vaccine dose (58).

Findings from this study support the first hypothesis, however it is likely, in our view that both theories have a part to play. At later stages, increased infectivity and virulence may have contributed to both increasing numbers of hospital admissions for acute COVID-19 and reduced proportions and absolute numbers of PIMS-TS patients.

This study confirms previous reports on PIMS-TS disproportionately affecting minority ethnic groups (23, 45, 60, 61). Notably, this contrasted with acute COVID-19 which reflected the regional composition of ethnic groups (62). While the exact reason for the predominance of ethnic minorities in PIMS-TS remain unknown, it suggests the involvement of genetic factors.

As mentioned above, children admitted for PIMS-TS were older children when compared to acute COVID-19, which is in line with previous reports (45, 46). However, young age also represents a possible risk factor for severe COVID-19 in children. Indeed, 39.8% (64/161) of patients admitted for COVID-19 in this study were younger than 1 year. This study also highlighted that children <1 year of age are at particular risk of becoming hospitalised with severe acute COVID-19. Notably, children >6 months have only recently become eligible for vaccination, but only if they are of an at-risk group (63). Given the risk profile, universal access to SARS-CoV-2 vaccines for infants, possibly through its incorporation into the childhood vaccination schedule, may prevent hospitalisation and even deaths in future wave outbreaks. Currently, the COVID-19 vaccine is not licenced for neonates, leaving this group highly vulnerable to severe acute infection. Thus, pregnant women should not only be encouraged to receive vaccines due to the increased risk of developing severe disease themselves, but also to allow effective maternal antibody transfer that may potentially protect infants in the crucial first 6 months of life (64, 65). As an alternative, in young children and infants who have not received vaccination, the use of neutralising monoclonal antibody products may incur survival benefit during severe acute COVID-19 disease (66), but more robust safety and efficacy data is required for its routine use.

Another factor associated with admission for COVID-19 is the presence of pre-existing comorbidities. While, in this study and others, only a few PIMS-TS patients (here 4%) had reported comorbidities (17, 23, 67, 68), 42.2% of COVID-19 patients had underlying disease (15, 69). Admissions for acute COVID-19 associate with a history of premature birth (13, 45), young age (<12 months) at infection (45, 61, 69, 70), type 1 diabetes (70), neurologic disorders (13, 45, 69, 70), cardiac disease (13, 45, 69, 70), respiratory disease (13, 45, 69), gastro-intestinal disease (45), malignancy (69), immunosuppression (16), chromosomal disorders including trisomy 21 (69), chronic kidney disease (69) and obesity (61). This suggests that the presence of comorbidities should be another key factor informing decisions on who to target in vaccination programmes and with therapies for non-hospitalised acute COVID-19 infections.

Patients admitted to hospital with PIMS-TS exhibited acute and severe disease but responded to anti-inflammatory and supportive treatment, which was in agreement with previous reports from us and others (45, 46, 61). When compared to COVID-19 patients, PIMS-TS patients had a greater length of admission (46), and an increased likelihood of critical care admission (45, 46). As with other studies, PIMS-TS associated with more gastrointestinal and neurological manifestations as well as mucocutaneous features (23, 45, 46, 60, 61, 71). Patients admitted for COVID-19 were, overall, less acutely unwell at admission. Nonetheless, 7/161 (4.3%) COVID-19 patients did not survive. In line with previous reports, and similar to the adult patient population, mortality in acute COVID-19 was associated with co-morbidities (15, 72). These observations are of relevance when, e.g., considering pre-hospitalisation treatment in high-risk patients (below).

At admission, the differentiation between acute COVID-19 and PIMS-TS can be challenging. Notably, while usually acute infection with SARS-CoV-2 precedes the onset of PIMS-TS by several weeks, some patients with PIMS-TS can remain PCR positive. This may be associated with persistent primary infection (73), or be the result of re-infection. In line with its hyper-inflammatory nature and previously reported features of “cytokine storm” (25, 74), PIMS-TS patients displayed markedly elevated markers of inflammation (white cell counts, neutrophils, C-reactive protein) (23, 45, 46, 60, 61, 71, 75). When compared to COVID-19, PIMS-TS associated with prominent lymphopenia and thrombocytopenia (23, 45, 46, 60, 61, 71, 75). However, severe COVID-19 may also present features of cytokine storm, such as pro-inflammatory cytokines and acute phase reactants, lymphopenia, and coagulopathy (76). Applying PLS-DA and VIP scoring, we identified CRP elevation and reduced platelet numbers as laboratory parameters with the highest contribution to differentiating PIMS-TS from acute COVID-19. Hyponatraemia, which was more common among PIMS-TS patients (46), may, as in “historic” Kawasaki disease, be explained by inappropriate antidiuretic hormone release (77). While these phenotype-associated features may aid in discriminating PIMS-TS from COVID-19, they are overlapping and require validation in larger independent patient cohorts, including further differential diagnoses such as other viral infections and bacterial sepsis (23, 45, 46, 60, 61, 71, 75).

In CYP, the evidence for treatment choices and associated risk is less robust when compared to the adult COVID-19 patient cohort (78, 79). At Alder Hey, treatment decisions were made by a multi-disciplinary team (including members from the infectious disease, rheumatology, general paediatrics, cardiology, and respiratory departments), which aided with balancing the relative effectiveness and safety of therapeutic options with changing virus variants over time. Treatment of acute COVID-19 with glucocorticosteroids was recommended from September 2020 in the UK, and was the mainstay of treatment for children requiring oxygen therapy (78). Remdesivir was licensed in July 2020, tocilizumab in December 2021 (78). Thus, these agents were only used in a minority of COVID-19 patients included here. For pre-hospitalisation treatments to prevent severe COVID-19, Ronapreve (August 2021) and Sotrovimab (December 2021) were available for CYP aged 12 years old and older weighing at least 40 kg (78). Among PIMS-TS patients in this cohort, 60% received IVIG and intravenous glucocorticosteroids, and 4% received tocilizumab or anakinra, to which they had been randomised through the RECOVERY trial (80).

While adding to the understanding of phenotypical, laboratory and prognostic differences between PIMS-TS and COVID-19, this study has limitations. In this single-centre retrospective study, virus variant data was not available for the majority of patients and had to be estimated based on regional surveillance data over time. Medical records did not always contain ethnicity data, SARS-CoV-2 vaccination status or body mass index. Thus, we were unable to determine whether for e.g., obesity was a risk factor. Laboratory tests were more frequently performed in PIMS-TS patients when compared to acute COVID-19 patients, and patients with milder acute COVID-19 were less likely to undergo laboratory testing. Only complete cases were compared in univariate analysis of laboratory tests, and multi-variate analysis of peak laboratory tests, possibly allowing bias.

## 5. Conclusions

Both alterations to the SARS-CoV-2 spike protein and increasing seroconversion likely contributed to decreasing admissions for PIMS-TS over time. Especially in times with low PIMS-TS case numbers, its diagnosis and differentiation from acute COVID-19 can be challenging. Clinical features, including mucocutaneous and enteric symptoms, thrombocytopenia, and a significantly elevated CRP associate with PIMS-TS, which may aid diagnosis, and rapid commencement of effective therapy. Patients with PIMS-TS can be acutely ill but usually respond to anti-inflammatory and supportive measures. While a smaller proportion of paediatric patients with acute COVID-19 require intensive care admission, 4.3% did not survive. Severe disease and mortality in COVID-19 are associated with young age (infancy) and pre-existing health conditions, including cardiovascular, mitochondrial, and neurological disease. Thus, vaccine programmes should include young age groups and especially target children with pre-existing diseases.

## Data Availability

The original contributions presented in the study are included in the article/Supplementary Material, further inquiries can be directed to the corresponding author.
