# Supplementary material for: Demographic, clinical and laboratory differences between paediatric acute COVID-19 and PIMS-TS—results from a single centre study in the UK
Source: Front Pediatr. 2023 Nov 10;11:1219654. doi: 10.3389/fped.2023.1219654 (PMC10667694; doi:10.3389/fped.2023.1219654)
Supplement: Supplementary file 1 [file Datasheet1.pdf]

Supplementary Data

<u>Supplement Table 1:</u> Breakdown of comorbidities for acute COVID-19 patients

Comorbidities	Total (*n = 161*)	Breakdown of comorbidities for acute COVID-19 patients*	*n = 161*
**Neurological and neurodevelopmental**	15/161	Cerebral palsy	4/161
		Autoimmune encephalitis	3/161
		Hydrocephalus	2/161
		Paraventricular leukomalacia	2/161
		Drug-resistant epilepsy	1/161
		Haemorrhagic necrotising encephalitis	1/161
		Methyl-CpG-binding protein 2 Xq28 duplication syndrome	1/161
		Midline glioma	1/161
		Necrotising myopathy	1/161
		Spinal muscular atrophy	1/161
**Cardiovascular**	25/161	Congenital cardiac defect	17/161
		Hypertension	4/161
		Supraventricular tachycardia on medication	2/161
		Shone's syndrome	2/161
		Aortic and mitral valve replacement	1/161
		Cardiomyopathy	1/161
		Long QT syndrome	1/161
**Metabolic/endocrine**	14/161	Hypothyroidism	3/161
		Mitochondrial disease	2/161
		Type 1 Diabetes Mellitus	2/161
		Type 2 Diabetes Mellitus	2/161
		Congenital hyperplasia due to 3-beta hydroxysteroid dehydrogenase deficiency	1/161
		Hyperinsulinaemic hypoglycaemia	1/161
		Hypoparathyroidism	1/161
		Multiple acyl-CoA dehydrogenase deficiency	1/161
		Pituitary stalk interruption syndrome	1/161
**Respiratory**	16/161	Chronic lung disease	6/161
		Asthma	3/161
		Obstructive sleep apnoea	2/161
		Bronchiolitis obliterans	1/161
		Intermittent aspiration pneumonia	1/161
		Tracheostomy	1/161
		Thoracic dystrophy causing oxygen requirement	1/161
		Long-term non-invasive ventilation	1/161
**Primary immunodeficiency**	2/161	Hypogammaglobulinaemia	2/161
**Secondary immunodeficiency**	16/161	Chemotherapy	7/161
		Long-term immunosuppression	9/161
**Oncological**	8/161	Haematological	5/161
		Ewing’s sarcoma	1/161
		Midline glioma	1/161
		Rhabdomyosarcoma	1/161
**Renal**	12/161	Chronic kidney disease (of which 5/161 were on regular dialysis)	8/161
		Renal transplant	3/161
		Congenital nephrotic syndrome	1/161

		Dysplastic kidney	1/161
		IgA nephropathy	1/161
		Polycystic kidney disease	1/161
**Liver**	3/161	Biliary atresia & Kasai procedure	1/161
		Liver transplant	1/161
		Polycystic liver disease	1/161
**Other**	16/161	Multisystem genetic disorders	7/161
		Gastrointestinal disease	7/161
		Systemic vasculitis	2/161
		Ulcerative colitis	2/161
		Factor VIII deficiency	1/161
		Sickle cell disease	1/161

***Comorbidities included under each category for COVID-19.** Some children may have had several comorbidities under one category but were only recorded in that category once. e.g. a child with congenital heart disease and hypertension is recorded once under cardiac but both comorbidities are listed above. Only two comorbidities were recorded in the PIMS-TS group (asthma 2/50).

<u>Supplement Table 2:</u> Peak laboratory test results

Blood tests	PIMS-TS Median (IQR)	*PIMS-TS (n)*	COVID-19 Median (IQR)	*COVID-19 (n)*	p value
Inflammatory state					
Lowest WBC *10^9/L	6.8 (5.67)	*48/50*	4.78 (4.08)	*80/161*	0.0019
Highest WBC *10^9/L	17.4 (7.02)	*48/50*	9.47 (11.4)	*80/161*	<0.001
Lowest neutrophils *10^9/L	5.21 (3.89)	*48/50*	1.94 (2.95)	*80/161*	<0.001
Highest neutrophils *10^9/L	14.2 (8.17)	*48/50*	6.56 (8.96)	*80/161*	<0.001
Lowest lymphocytes *10^9/L	0.65 (0.587)	*48/50*	1.19 (1.37)	*80/161*	<0.001
Highest lymphocytes *10^9/L	2.55 (1.59)	*48/50*	2.77 (3.04)	*80/161*	0.440
Highest CRP mg/L	231 (108)	*50/50*	19.6 (87.7)	*89/161*	<0.001
Highest ferritin ng/mL	812 (1201)	*35/50*	458 (1466)	*16/161*	0.386
Highest triglycerides mmol/L	2.3 (0.795)	*15/50*	2.4 (2.01)	*11/161*	1
Coagulation					
Lowest platelets *10^9/L	112 (76)	*49/50*	236 (146)	*80/161*	<0.001
Highest platelets *10^9/L	384 (225)	*49/50*	337 (202)	*80/161*	0.196
Highest INR	1.19 (0.24)	*37/50*	1.41 (0.68)	*35/161*	0.1956
Lowest fibrinogen g/L	3.3 (1.4)	*33/50*	1.6 (1.5)	*27/161*	<0.001
Highest fibrinogen g/L	5.3 (1.2)	*33/50*	3 (1.95)	*27/161*	<0.001
Highest D-dimer ng/mL	4863 (4812)	*33/50*	3206 (8968)	*16/161*	0.301
Lowest Hb g/L	95 (16.5)	*48/50*	95 (22)	*80/161*	0.8189
Renal function					
Lowest sodium mmol/L	131 (6)	*49/50*	135 (4.5)	*91/161*	<0.001
Highest urea mmol/L	7.9 (6.4)	*49/50*	4.5 (4.17)	*91/161*	<0.001
Highest creatinine mmol/L	56 (43)	*49/50*	40 (31)	*91/161*	<0.001
Liver function					
Highest bilirubin µmol/L	11 (7.5)	*43/50*	9.5 (17)	*65/161*	0.803
Highest ALT iu/L	51 (50)	*43/50*	54.5 (92.8)	*65/161*	0.571
Highest AST iu/L	48 (66)	*43/50*	64.5 (109)	*65/161*	0.183

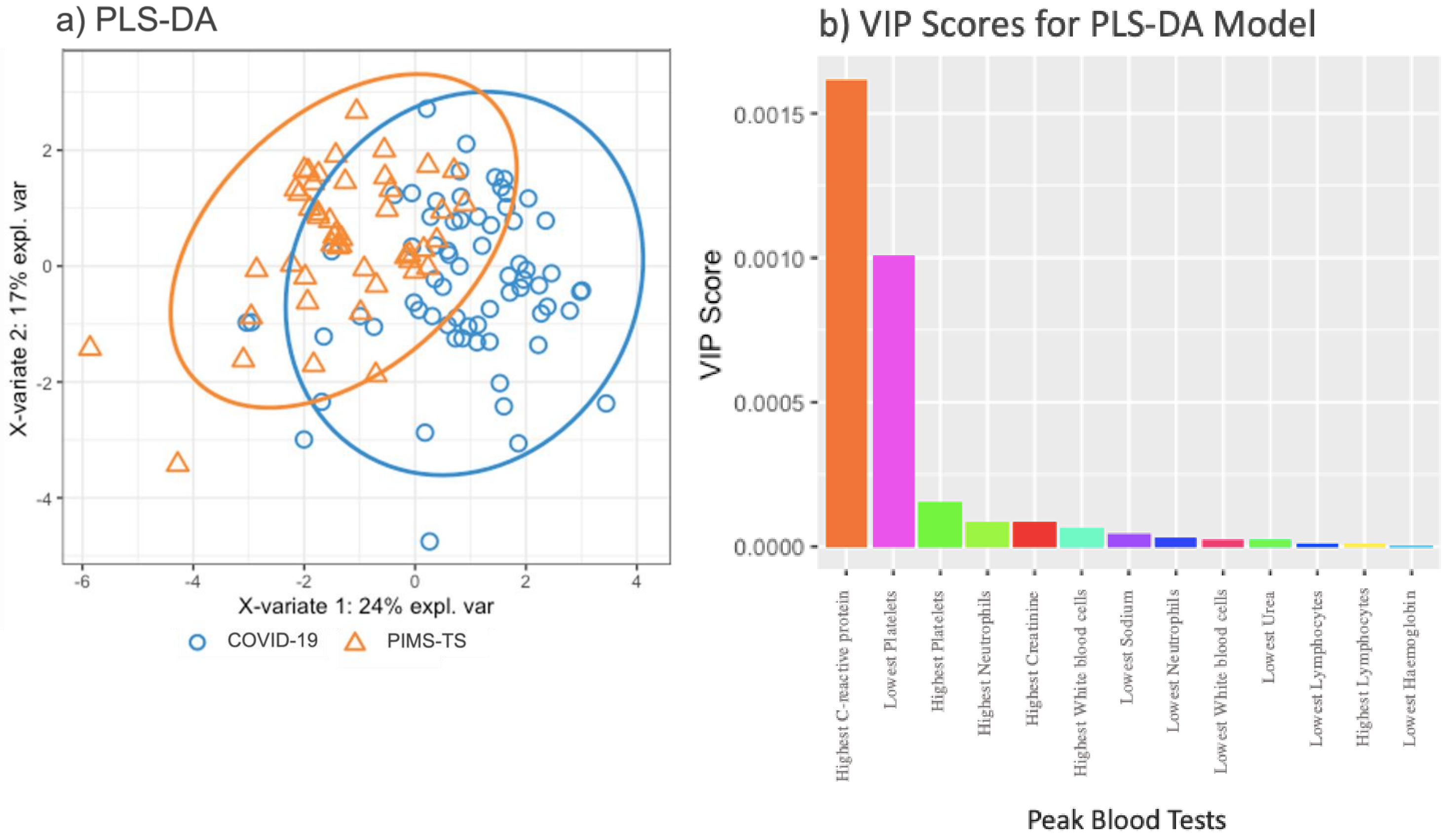

Supplement Figure 1: Partial least squares-discriminant analysis (PLS-DA) in PIMS-TS *(n=47)* and COVID-19 *(n=68)* patients during entire admission. Blood tests included were haemoglobin (lowest), platelets (highest and lowest), white blood cells (highest and lowest), neutrophils (highest and lowest), lymphocytes (highest and lowest), C-reactive protein (highest), sodium (lowest), urea (highest), creatinine (highest). A) PLS-DA analysis showing individual samples with the confidence ellipses of PIMS-TS and COVID-19. B) VIP analysis showing variable importance plots of blood tests included in the PLS-DA model.
